# Regulatory withdrawal of medicines marketed with uncertain benefits: the bevacizumab case study

**DOI:** 10.1186/s40545-015-0046-2

**Published:** 2015-10-19

**Authors:** Agnes Vitry, Tuan Nguyen, Vikky Entwistle, Elizabeth Roughead

**Affiliations:** Quality Use of Medicines and Pharmacy Research Centre, Sansom Institute, School of Pharmacy and Medical Sciences, University of South Australia, GPO Box 2471, Adelaide, SA 5001 Australia; Health Services Research Unit, University of Aberdeen, Aberdeen, AB25 2ZD United Kingdom

**Keywords:** Pharmaceutical policy, Managed entry agreement, Medicine subsidization, Coverage with evidence development

## Abstract

**Background:**

Withdrawal of conditional regulatory approval or subsidization of new medicines when subsequent evidence does not confirm early trial results may not be well understood or accepted by the public.

**Objectives:**

We present a case study of the US Food and Drug Administration (FDA)’s decision to withdraw the indication of bevacizumab for the treatment of advanced breast cancer and include an analysis of the reactions of stakeholders with a view to identifying opportunities for improving risk management for new medicines with conditional approval or funding.

**Methods:**

We drew on a range of information sources, including FDA documents, medical journals and media reports, to describe the evidentiary basis of the FDA decisions. We analysed the reactions and perspectives of the stakeholders.

**Results:**

In 2008 bevacizumab was granted conditional approval for treatment of advanced breast cancer by the FDA pending submission of supplementary satisfactory evidence. In 2011 the FDA decision to withdraw the indication was met with a hostile reaction from many clinicians and cancer survivors. There were different interpretations of the therapeutic value of bevacizumab with strong beliefs among cancer survivors that the medicine was effective and potential harm was manageable. High expectations of the public may have been encouraged by overly positive media reports and limited understanding by the public of the complexity of the scientific evaluation of new medicines and of the regulatory processes.

**Conclusions:**

Improving understanding and acceptance of approval or coverage schemes conditional to evidence development may require the development of risk management plans by regulatory and funding institutions. They may include a range of strategies such as requirements for formal patient acknowledgment of the conditional availability of the medicine, ‘black-triangle’ equivalent labels that identify full approval is based on pending evidence, and ongoing communication with the media, public and health professionals.

## Introduction

In the United States, the Food and Drug Administration (FDA) has implemented an accelerated approval program for medicines that appear to provide a benefit for serious or life-threatening illnesses lacking satisfactory treatments [1]. Under this program, medicines are given a conditional approval based on clinical trial data that suggest efficacy but are not sufficient to permit full approval. Full approval depends on subsequent confirmatory clinical trials. Some countries with national public health insurance systems, including Australia, Canada, Italy and the United Kingdom, have introduced similar ‘coverage-with-evidence development’ schemes in which approved medicines may be subsidised pending the later submission of satisfactory research data [2].

These new schemes are attractive for policymakers as they may temporarily resolve tensions between the objectives of (a) maintaining efficacy, safety and cost-effectiveness standards when granting approval or coverage of new medicines and (b) meeting public and industry’s expectations for access. However, withdrawal of conditional approval or funding of medicines when the additional research does not confirm early favourable results may not be well understood or accepted by patients and health professionals, and regulatory and funding agencies need to prepare for this possibility.

There have been, as yet, few examples of withdrawals of medicines for reasons of uncertain efficacy after conditional approval. In 2011 the FDA announced its decision to withdraw the marketing approval of bevacizumab (Avastin®) for the treatment of advanced breast cancer. Bevacizumab had been approved for this indication under the FDA accelerated approval program in 2008. In this paper, we consider this case study as the first example of an indication being withdrawn due to uncertain efficacy in a highly sensitive political context, the therapeutic management of advanced breast cancer. The withdrawal was highly contested, and this is a case from which other countries with schemes for regulatory approval or coverage conditional on evidence development could usefully learn. We analysed both the basis of the FDA’s decisions and stakeholders’ reactions to them with a view to informing the development of risk management plans conditional approval or funding schemes.

## Methods

A documentary analysis was undertaken based on a range of documentary sources relating to bevacizumab published between 2005 and 2012. From the FDA website documents were retrieved related to the Oncologic Drug Advisory Committee (ODAC) meetings, transcripts and public submissions to the FDA, and the final 69-page document by Margaret Hamburg, the FDA commissioner explaining the reasons for bevacizumab withdrawal. Medical journal articles were identified through a literature search on the Web of Knowledge website. Media reports were obtained from Drug Information Daily newsfeed and Factiva news media database using ‘bevacizumab’ or ‘Avastin’ as search terms.

An analytical framework was developed with a focus on three main themes for data analysis: (1) rationale of FDA’s decisions for granting and revoking approval of bevacizumab for the treatment of advanced breast cancer; (2) stakeholders’ involvement and reactions to the decisions; and (3) implications for future risk management strategies. Specifically, government documents and medical journal articles were used to retrace the regulatory approval history of bevacizumab. A descriptive analysis of the evidentiary basis of the FDA decisions was conducted considering the types of scientific uncertainties that generated conflicting interpretations. Media reports, medical journal articles and government documents were utilised to examine the reactions of key stakeholders, including patients, consumer organisations, the pharmaceutical industry, health professionals and the media, to the FDA withdrawal process. These three sources of information provided complementary insights on the debates, both within the medical community and among the public at large, which took place over that period. Insights from the first two themes informed discussion of strategies that could be used to improve the public understanding and acceptability of schemes for regulatory approval or coverage conditional on evidence development.

## Results

### Background and history of events

Bevacizumab is a recombinant humanised monoclonal antibody to vascular endothelial growth factor (VEGF) marketed by Genentech as Avastin®. Bevacizumab inhibits the binding of VEGF to its receptors on the surface of endothelial cells, thereby reducing the vascularisation of cancerous tumours and inhibiting their growth.

In 2004, bevacizumab was first approved by the FDA as treatment for metastatic colorectal cancer. It then gained approval for other indications including non-small cell lung cancer, renal cell carcinoma, and glioblastoma [3]. In February 2008, bevacizumab was approved for first-line treatment of metastatic breast cancer under the FDA’s accelerated approval program (Table 1). The approval was based on the results from a single study, the E2100 trial, published in the *New England Journal of Medicine* (NEJM) journal in 2007 [4]. This trial showed an increase in median progression-free survival in women with advanced breast cancer treated with bevacizumab in combination with paclitaxel but no increase in overall survival compared to those treated with paclitaxel alone [4] (Table 2). Reprints of the NEJM article were actively used by Genentech, the makers of bevacizumab, to promote the drug to physicians [5] and Genentech estimated that about 9000 breast cancer patients in the United States had been treated with ‘off-label’ bevacizumab (i.e. outside marketing authorisation) at the time of the FDA’s approval in 2008 [6].

**Table 1:** History of regulatory events

Date	FDA decisions
26 February 2004	FDA approval of bevacizumab as first-line treatment for metastatic colorectal cancer in combination with intravenous 5-fluorouracil
20 June 2006	FDA approval of bevacizumab as second-line treatment for metastatic colorectal cancer
5 December 2007	ODAC voted 5 to 4 against the recommendation bevacizumab for treatment of metastatic breast cancer in combination with paclitaxel
22 February 2008	FDA granted accelerated approval of bevacizumab in combination with paclitaxel for the treatment of metastatic breast cancer
20 July 2010	ODAC voted 12 to 1 revoking approval of bevacizumab for the treatment of metastatic HER2 negative breast cancer
16 December 2010	FDA initiated the withdrawal process
16 January 2011	Genentech requested an hearing
28–29 June 2011	FDA Oncologic Drugs Advisory Committee hearing recommended the indication withdrawal
18 November 2011	FDA withdrew officially the indication

**Table 2:** Efficacy results of bevacizumab trials [28]

Trial	Line in therapy	Trial years	Number patients enrolled	Comparison	Progression-free survival		Overall survival	
					Median difference (months)	Hazard Ratio (95 % confidence interval, P value)	Median difference (months)	Hazard Ratio (95 % confidence interval, P value)
AVF2119g trial [63]	Second line (previously treated with anthracycline and taxane)	2000–2002	462	Bevacizumab plus capecitabine versus capecitabine	0.7	0.98 (0.77–1.25, *P* < 0.86)	0.6	1.05 (0.86–1.30, *P* < 0.63)
E2100 trial [4, 10]	First line	2001–2004	722	Bevacizumab plus paclitaxel versus paclitaxel	5.5	0.48 (0.39–0.61, *P* < 0.0001)	1.7	0.87 (0.72–1.05, *P* = 0.137)
AVADO trial (BO17708) [13, 28]	First line	2006–2007	736	Bevacizumab plus docetaxel versus docetaxel	0.9	0.62 (0.48–0.79, *P* = 0.0003)	- 1.7	1.00, 0.76–1.32, *P* = 0.98)
RIBBON-1 (AVF3694g) [14, 28]	First line	2005–2007	622	Bevacizumab plus taxane/anthracycline versus taxane/anthracycline	1.2	0.64 (0.52–0.80, *P* < 0.0001)	Not reported	1.11 (0.86–1.43, *P* = 0.44)
		2005–2007	615	Bevacizumab plus capecitabine versus capecitabine	2.9	0.69 (0.56–0.84, *P* =0.0002)	2.9	0.88 (0.69–1.13, *P* = 0.33)
RIBBON-2 (AVF3693g) [28]	Second line	2006–2008	684	Bevacizumab plus taxanes, capecitabine or gemcitabine versus taxanes, capecitabine or gemcitabine	2.1	0.78 (0.64–0.93, *P* = 0.0072)	Non significant difference	

The official review of bevacizumab for metastatic breast cancer by ODAC was less positive than the NEJM paper. It highlighted several methodological shortcomings of the E2100 trial, including the use of progression-free survival as an endpoint and the lack of blinding [7]. Progression-free survival has not been shown convincingly to be an appropriate surrogate endpoint for breast cancer or to be predictive of overall survival [8]. The lack of blinding meant that knowledge of which drug(s) individual patients were receiving could have influenced judgements of their responses to treatment. An independent, blinded review of radiological and clinical data of all patients in the E2100 trial was therefore required by the FDA. Although this confirmed that the addition of bevacizumab to paclitaxel resulted in a statistically significant improvement in progression-free survival (hazard ratio 0.48, 95 % CI 0.39 to 0.61; *P* < 0.0001) [9], the estimate of the magnitude of the effect lacked reliability because of incomplete data (10 % of patients), loss to follow-up (34 % patients) and lack of consistency in determination of radiologic disease progression [7]. ODAC also highlighted important safety issues with bevacizumab including a 20.2 % increase in grade 3–5 toxicity (including hypertension, sensory neuropathy, thromboembolism, gastrointestinal perforation, haemorrhage) and 1.7 % incidence of treatment-related death in the bevacizumab plus paclitaxel arm compared to 0 % the paclitaxel arm [7, 10].

Based on all this evidence, ODAC, which comprised seven experts in the field of oncology or statistics, and two consumer representatives, voted five to four in an open voting process not to recommend approval on the question of whether the data provided were ‘*sufficient to establish a favourable risk/benefit analysis for the use of bevacizumab plus paclitaxel for first-line treatment of patients with metastatic breast cancer*” [11].

Despite the ODAC vote, the FDA granted approval contingent on the results of additional studies in February 2008. This decision generated great interest for both financial and health reasons. It markedly benefited the drug maker’s potential market and sales revenue, and Genentech’s stock rose by more than 8 % in after-hours trading [12]. The FDA decision was also perceived as clinically controversial. Some health professionals and a patient advocacy group, the National Breast Cancer Coalition Fund, expressed concern about a lowering of the FDA standards for medicine approval [12]. Other experts welcomed the decision because they believed that it was ‘*a matter of time before a survival benefit is documented*’ [6].

Almost two and a half years later, in July 2010, the ODAC convened to re-evaluate the approval and examined the results of two additional clinical trials [13, 14] (Table 2) and voted 12 to one to recommend removing the indication of bevacizumab for metastatic breast cancer [15]. The two new studies did not demonstrate a difference in overall survival and showed smaller improvement in progression-free survival than in the original E2100 trial. None of the studies demonstrated an improvement in quality of life and all showed an increased risk of serious adverse effects including gastrointestinal perforation and severe bleeding (Table 3). The overall proportion of treatment-related deaths was similar (1.8 %) in both bevacizumab and control groups [16].

**Table 3:** Pooled safety results of bevacizumab trials for first-line treatment of advanced breast cancer (E2100, AVADO, RIBBON-1 y

Severe or life-threatening or fatal adverse events	Pooled chemotherapy (*n* = 982)	Pooled bevacizumab plus chemotherapy (*n* = 1679
	%	%
Any	23	37
Sensory neuropathy	7.1	10
Hypertension	1.2	9
Febrile neutropenia	3.5	6.5
Venous thromboembolic event	3.8	2.8
Proteinuria	0	2.3
Arterial thromboembolic event	0.3	1.6
Left ventricular systolic dysfunction	1.2	1.5
Hemorrhage	0.4	1.5
Abnormal tissue repair	0.8	1.7
Wound dehiscence	0.3	0.8
Fistula	0.3	0.5
Gastrointestinal perforation		0.5
Reversible posterior leukoencephalopathy syndrome	0	<0.1

In December 2010, the FDA announced it would start withdrawing the indication. Around 17,000 women with advanced breast cancer were being treated with bevacizumab at that time, and financial analysts estimated that a withdrawal of FDA approval for breast cancer could cost Genentech nearly $1 billion in sales based on previously projected figures [17]. Genentech filed an opposition petition requesting an administrative hearing.

In June 2011, there was a two-day hearing which involved ODAC experts, experts nominated by Genentech and permitted members of the public to provide oral testimony. Electronic or written comments on the FDA’s proposal to withdraw approval were also invited [18].

Around 450 public submissions were sent to the FDA during the hearing period, mostly by consumers asking the FDA to maintain the indication as their perception was the drug had benefited themselves or close friends and family [19]. In the first part of the hearing, 35 members of the public provided their views. They included survivors of advanced breast cancer and representatives from consumer organisations including ‘Facing Our Risk of Cancer Empowered’(FORCE, representing people living with a genetic mutation or hereditary cancer risk), the Abigail Alliance for Better Access to Developmental Drugs, the Ovarian Cancer National Alliance, breastcancer.org, Marti Nelson Cancer Foundation, Colon Cancer Alliance, Kidney Cancer Association, Cancer Support Community. Most consumer groups urged the FDA to keep bevacizumab available [20]. Survivors ascribed their survival and current quality of life to bevacizumab and called themselves ‘*super-responders*’. There was no comment from the public on the possibility of predicting in advance how responders might be distinguished from non-responders or whether responders may constitute a minority of women. This question was acknowledged as unresolved during the following scientific discussions of ODAC as no discriminatory biological or genetic marker for predicting the efficacy of bevacizumab had been identified in the clinical trials.

Concerns on the safety of bevacizumab were rarely raised by members of the public during the hearing, and when they were it was usually to say that the adverse effects of bevacizumab were mild or manageable. Only one woman from SHARE leaders, a group of cancer survivors, said that ‘*for every woman here testifying, there are other women who we know – a member of our group who bled out of every orifice of her body…another woman…who had a brain haemorrhage. So those people don’t come to testify’*. Among the few consumer voices that supported bevacizumab withdrawal was that of Christine Brunswick, the vice president of the National Breast Cancer Coalition and breast cancer survivor who stated that ‘*This decision can't be driven by anecdotes. It must be driven by scienc*e’. At the end of the hearing, ODAC voted 6–0 in favour of removing the indication and the FDA finally withdrew its approval in November 2011, three and a half years after the initial conditional approval.

The FDA withdrawal process prompted reactions from patients, consumer organisations, health professionals, healthcare policy makers and the pharmaceutical industry, and the decision generated major media coverage in newspapers and on television. Some patients were very upset when the vote was announced with one woman accusing the FDA committee of ‘*killing 17,000 women with one vote*’ [21]. But criticism was not based solely on patients’ beliefs in bevacizumab’s benefits. Freedom of Access to Medicines, a project from The Abigail Alliance that lobbies for wider access to developmental cancer drugs, questioned the right of the FDA ‘*to make a decision that should be left to a woman and her doctor*’ [22]. The FDA was also accused of taking cost into consideration [22].

Some consumer organisations feared that insurers would not pay for the cost of bevacizumab after the approval withdrawal. Susan G. Komen from the Cure, the US largest breast cancer organization, declared that “*as a patient advocacy organization, we want to ensure that women who are successfully using Avastin today continue to have access to the drug, and that their treatment be covered by third-party payers*” [23]. Medicines without FDA regulatory approval may still be prescribed and used by patients ‘off-label’, i.e. for indications outside the marketing authorizations, however, the extent to which public or private health insurers may cover off-label use may vary substantially [24]. Insurers initially appeared to respond to these consumer concerns. The day after the FDA hearing, the Centers for Medicare and Medicaid Services (CMS), which determines how products are reimbursed under Medicare, the largest public health insurance in the US, announced that they would ‘*continue to cover the drug for breast cancer as long as doctors are prescribing it*’ [25]. The guideline committee of the National Comprehensive Cancer Network (NCNN), a consortium of 21 cancer centers, whose guidelines are one of the main compendia used by American health insurers to inform their coverage decisions [26], voted 24 to 0 with one abstention to continue recommending bevacizumab in combination with paclitaxel as an appropriate treatment option for metastatic breast cancer for the reason that it ‘*improves time to progression and response rates but does not improve overall survival’* [27]. It was noted that nine of the 27 members of the Breast Cancer Panel in charge of the NCNN guidelines on management of breast cancer received financial support from Genentech, the company manufacturing bevacizumab [28]. The extent to which this had an impact, if any, on the reluctance of the NCCN to change their guidelines in response to the FDA withdrawal of the indication is unknown. Three months later, however, Blue Shield of California became the first large insurance company to announce it would end payments for bevacizumab in the management of advanced breast cancer [29].

### Health professional and medical organisation responses

The FDA withdrawal process was heavily debated in medical journals. An editorial in the *Journal of Clinical Oncology* supported the FDA’s decision, stating that “*the outcomes were arguably not clinically compelling*” [30]. Ralph D’Agostino, a professor of mathematics who served on ODAC at the time of FDA’s initial approval of bevacizumab, argued in the *New England Journal of Medicine* that progression-free survival was not an acceptable primary endpoint for approval of first-line therapies in cancer and ‘*if its use becomes standard for accelerated or even final approval, it will be more difficult, if not impossible, to obtain solid data on overall survival*’ [31]. An oncologist who served as an ODAC member at the FDA hearing that considered the withdrawal decision declared ‘*we did not want people to be hurt by a drug that does not work that well. We do not want to provide false hope*’ [32]. In contrast, Dr Milton Wolf, a radiologist, wrote an article for the conservative *Washington Times* entitled ‘*The FDA’s one-man death panel*’ and claimed that the ‘*FDA denies Americans access to life-saving drugs*.’ He described the cost and complexity of the FDA’s processes as ‘*regulatory barriers*’ that impede the access to innovative medicines in the US.

The opinions of health professionals on the FDA withdrawal decision were examined in an study conducted after ODAC’s recommendation to revoke approval of bevacizumab between September and December 2010 [33]. This email survey included 564 participants from across the world, mostly medical oncologists. About 50 % of the respondents said that, if the DFA cancelled approval, they would use bevacizumab in an off-label indication, mainly in patients with triple-receptor negative breast cancer. A small majority (52 %) agreed with the FDA decision to withdraw the indication on the ground that the benefits shown in the two additional studies with bevacizumab were not of the same amount observed in the initial E2100 trial but 48 % believed this was not a valid reason. Another study examined trends in use of bevacizumab for breast cancer in 122 oncology practices involving 570 oncologists in the US [34]. It found that the use declined by 37 % between May 2010 (just prior to the ODAC meeting revoking approval) and November 2010 (just prior to the start of the FDA withdrawal process) and by 63 % just prior to the FDA official withdrawal notice without concomitant changes to clinical guidelines or insurers’ coverage policies that may explain these trends.

### Media coverage

A study of 359 articles published in North American newspapers about bevacizumab and breast cancer before, during and after the FDA approval period showed that, prior to the FDA approval, the reports tended to present bevacizumab positively: 82 % of articles noted efficacy and only 23 and 24 % reported the lack of efficacy or side effects respectively [35]. The proportions of positive headline tone (36 % before approval, 18 % during approval, 9 % after approval, *p* = 0.0002) and positive article tone (42 %, 19 %, 15 %, *p* < 0.0001) declined with each study period. Industry representatives were more likely to be quoted prior to the approval than at later times (33 %, 23 %, 11 %, *p* = 0.014).

## Discussion

There is a number of international examples where the potential withdrawal of medicines which had been made available pending confirmatory scientific evidence was strongly opposed by pharmaceutical companies or consumers. The negative scientific findings of a costly patient access scheme for medicines for multiple sclerosis in the United Kingdom did not trigger any price reduction nor withdrawal of the medicines and this failure was at least partly attributed to unclear governance processes [36]. Still in the United Kingdom, the recent decision to withdraw 16 medicines covering 25 cancer indications from the Cancer Drug Fund, a scheme which gave cancer patients access to cancer medicines not approved yet by the National Institute for Clinical Excellence (NICE) [37] was opposed by patients and pharmaceutical companies [38]. However, it was announced later that the Cancer Drug Fund will become a ‘managed access’ fund for new cancer medicines which will be given ‘conditional approval’ by NICE and provided for a defined period whilst further evidence from real world use was collected [39].

As conditional approval or funding of medicines subject to the provision of satisfactory evidence is likely to expand in the future, the potential societal and human impact of withdrawal decisions has to be acknowledged and prospectively managed by regulatory and funding agencies. The public reaction to this case was perhaps particularly strong as breast cancer has a high public profile and bevacizumab had already achieved widespread use for other cancers. Nonetheless, there is certainly room for improving public understanding and acceptance of approval or coverage schemes conditional to evidence development. It will be contingent on regulators to develop and implement comprehensive risk management plans that include the potentiality of withdrawal of the conditionally approved indication. Insights from our analysis of the stakeholders’ responses to the FDA withdrawal allow us to examine the factors that may have fuelled some of the more emotional and acrimonious aspects of the debate and suggest a number of elements which may be incorporated in future risk management strategies.

### Conflicting interpretations of evidence

At the outset of this case, there were differences in interpretations of the research evidence with regards to the validity and clinical significance of the progression-free survival endpoint and the relative weighting of potential benefits and harms of bevacizumab. The purpose of regulatory decisions is to protect public health and to ensure collective safety and this may conflict with what individual patients want on some occasions. Some experts have expressed concerns that ‘*the plight of desperate patients may divert attention from the value to present and future patients and the society of ensuring the efficacy and safety of new treatments*’ [40]. Recently, the American Society of Clinical Oncology aimed to reach a consensus on what might constitute a “clinically meaningful outcome” target for clinical trials of new cancer medicines, for example a 4.5 to 6 months improvement over current overall survival for metastatic triple negative previously untreated metastatic breast cancer [41]. However, some cancer survivors’ representatives have argued that ‘*a month can be the equivalent of a year if you have limited life expectancy*’ [42], suggesting ongoing dialogue will be required and this debate is not readily resolvable.

### Patients’ beliefs about the effectiveness of bevacizumab

Other key issues that made management of the bevacizumab withdrawal difficult for the FDA were the convictions of breast cancer survivors that bevacizumab had “saved their life” and the broadly optimistic background beliefs of patients about the effectiveness of cancer treatments in general and bevacizumab in particular.

The voices of patients who favoured bevacizumab were certainly more widely and loudly heard than those of patients who might not have favoured it. There are several reasons for this. Survivors understandably often associate their survival with the treatment they received, and are often keen to speak up and support its continued availability. Patients who do less well and do not survive (and who were perhaps more likely to have experienced serious harms) are obviously less able to testify. Concerns about the lack or limited efficacy of bevacizumab in the majority of patients, and about the significant proportion of patients who may experience serious harms, were mostly absent from the public debate.

The strong patient reaction may have also stemmed from patients needing to maintain high expectations as a coping strategy or not to be seen to be ‘giving up’ and hastening death [43]. In this context, women with advanced breast cancer may have perceived the FDA withdrawal decision as taking away their only and last hope of survival.

Patients often over-estimate the efficacy of cancer treatments. A survey of US patients with advanced (metastatic) colorectal and lung cancer found that a large majority (69 % of patients with lung cancer and 81 % of those with colorectal cancer) did not understand that chemotherapy would not cure their cancer [44]. Patients’ beliefs on the therapeutic value of cancer medicines may be influenced by a complex range of clinical, social and commercial forces. They may be shaped by discussions with oncologists, which sometimes lead to false optimism [45].

Beliefs in the therapeutic value of bevacizumab may have been enhanced by its earlier approval in the management of advanced colorectal cancer. Bevacizumab received a lot of media attention when it was approved for this indication and was called a ‘*revolutionary*’ medicine [46]. The rapid uptake of bevacizumab in the first year after approval for colorectal cancer in the United States has been described as unprecedented for a cancer medicine [47]. People often incorrectly assumed that the benefits of targeted cancer medicines which are observed for a specific cancer type can be confidently extrapolated to other cancer types [48].

Approval of a medicine by the FDA, even if it is provisional, is likely to bolster public opinion of a product’s efficacy and safety. In a survey of about 3000 US adults, 39 % reported beliefs that the FDA approves only ‘extremely effective drugs’ and 25 % reported beliefs that the FDA approves ‘only drugs without serious adverse effects’ [49].

### Media reporting

High expectations about the value of new medicines are often fuelled by enthusiastic media reports, particularly early in the product life cycle when stories are initiated primarily by their commercial developers and associated clinical advocates [50]. Lay media value novelty and tend to emphasize clinical benefits rather than expressing uncertainties or discussing the potential harms of cancer medicines [51–53]. Information on the therapeutic value of new medicines may get ‘streamlined’, removing complexity and uncertainties [24]. Media reports may be informed by sensational statements or press releases issued by researchers, their institutions and the pharmaceutical industry which all stand to benefit in terms of reputation and profits [54]. The study of newspaper reports on bevacizumab and breast cancer [35] showed that bevacizumab was presented very positively prior to the FDA approval and this may have heartened perceptions among the public of the high therapeutic value of this medicine.

### Risk management planning, better communication and resetting public expectations

Improving public understanding and acceptance of approval or coverage schemes conditional to evidence development will require the concerted effort of different stakeholders. Medical journals and research institutions could try to present their findings on new medicines to the media in a way that increases the probability of balanced reporting by the mainstream media [54]. Specialised training for health journalists might also be useful. In this context, regulators such as the FDA have an important role to play in developing and implementing communication strategies as part of risk management plans that include contingency for future withdrawal of a conditionally approved indication.

Our analysis has identified a number of ways in which regulators and associated agencies could work proactively to promote the acceptability of justifiable withdrawals of approval, and to limit the distress these may cause to patients and the public.

Firstly, the difference in opinions between the initial negative recommendation of ODAC and the FDA approval decision could be seen, in hindsight, as an early warning signal for potential problems if subsequent approval or coverage needed to be reversed. Regulators could consider that, where votes are split for approval, they need to examine the reasons for the differences and consider using these in communication activities with the public, health professionals and the media. Risk management planning at the time of the initial approval or coverage decision may facilitate better understanding of the uncertainty of the clinical place in practice of the approved medicine. A number of documents were available on the FDA website, both at the time of bevacizumab approval and withdrawal, including full transcripts of hearings. However, the FDA documents are lengthy and difficult to navigate, making the information they contain difficult to access for lay audiences and also for health professionals [55]. Moreover, these documents may be challenging to find, which restricts diffusion of their findings [56]. Regulators need to ensure the information they provide is readily accessible and to diverse audiences.

Secondly, over and above the need to provide improved education of the public on the efficacy and safety of new medicines [57], more realistic patient and provider expectations need to be established, particularly where significant uncertainties with regards to efficacy of the medical technologies exist. It has been suggested that the FDA could provide a summative statement or a rating for each medicine approval to indicate the strength of clinical trial evidence used to determine safety and efficacy, allowing medicines approved on the basis of strong evidence to be distinguished from those approved on the basis of less strong evidence [58]. Medicines marketed under conditional approval or funding may also need to be flagged in some ways to alert or remind health professionals and patients of the limited nature of the evidence and preliminary nature of the approval– perhaps along the lines of the black box warnings and black triangles that have been developed to alert the public and providers to safety issues or limitations in safety information.

Thirdly, in some countries patient consent to continuing therapy based on achieving health improvements is a requirement of subsidisation. Similar strategies may be appropriate for conditional coverage schemes, where patients might be asked to acknowledge, when starting therapy, that there is still uncertainty about effectiveness or safety and to agree that continuing therapy may be dependent on subsequent evidence of efficacy or safety. Furthermore, it has been argued that it would be reasonable to require patients who access medical interventions of uncertain benefit to contribute data to ongoing evaluation [59]. Such schemes are currently developing as part of coverage with evidence development funding programs [2].

Finally, the potential for the media to promote unrealistic expectations of medicines highlights the need for an ongoing media communications strategy as part of the implementation of coverage with evidence development or provisional approval programs, particularly those that may be implemented over long time frames. In 2009, the FDA adopted a strategic plan for risk communication [60] and in 2010, the FDA Transparency Task Force published recommendations for making information about FDA activities and decision-making more readily available to the public in a timely manner and in a user-friendly format. However, this does not yet include risk management plans concerning conditional regulatory approvals. A targeted communication plan may include information on what regulatory and funding agencies know and do not know about new medicines that are marketed or funded. It may involve ongoing press releases and media briefings from regulators or insurers as part of a sustained communication strategy while awaiting evidence development. This plan may need to be adapted depending on the extent of product use.

Although it remains to be shown how these strategies may be applied in the setting of conditional approval or funding of medicines, some measures have already been implemented in practice. In 2011, the European Medicines introduced a new scheme, which imposed the use of an inverted black triangle symbol to improve public awareness of medicines subject to additional safety monitoring [61]. This scheme also applies to medicines granted conditional approval where the company is required to undertake additional safety studies [61]. In March 2015, the Australian Pharmaceutical Benefits Advisory Committee recommended that the revised Framework for the Managed Access Programme (MAP), where some medicines can be funded pending submission of more conclusive evidence of cost-effectiveness, should include ‘*a communication strategy, targeted at patients and prescribers, that clearly articulates the arrangement is central to any new or continued MAP (including the possibility that a drug may not be reimbursed indefinitely*’ [62]. It also recommended that ‘*patients who are prescribed a medicine listed as part of a MAP must understand the process and provide informed consent*’ [62].

## Conclusion

The withdrawal of conditional approval or conditional funding of new medicines due to uncertain efficacy may not be well accepted by the public for a number of reasons including the complexity of the scientific evaluation of new medicines and the development of unrealistic hopes fuelled by news media and the pharmaceutical industry. We have proposed strategies that may be considered by regulatory and funding agencies in the development of risk management plans in the future. They involve targeted, comprehensible and ongoing communication with the public and the media, specific labelling or marking of the medicines, and possibly formal patient acknowledgement of the nature and the risks of conditional approval.
